# Sexual crossing of thermophilic fungus *Myceliophthora heterothallica* improved enzymatic degradation of sugar beet pulp

**DOI:** 10.1186/s13068-016-0460-y

**Published:** 2016-02-20

**Authors:** Maria Victoria Aguilar-Pontes, Miaomiao Zhou, Sjors van der Horst, Bart Theelen, Ronald P. de Vries, Joost van den Brink

**Affiliations:** Fungal Physiology, CBS-KNAW Fungal Biodiversity Centre, Utrecht, The Netherlands; Fungal Molecular Physiology, Utrecht University, Utrecht, The Netherlands; Yeast and Basidiomycete Research, CBS-KNAW Fungal Biodiversity Centre, Utrecht, The Netherlands

**Keywords:** *Myceliophthora heterothallica*, Sexual crossing, Progeny, Plant biomass, Sugar beet pulp, Saccharification, CAZy enzymes, Acetyl xylan esterase

## Abstract

**Background:**

Enzymatic degradation of plant biomass requires a complex mixture of many different enzymes. Like most fungi, thermophilic *Myceliophthora* species therefore have a large set of enzymes targeting different linkages in plant polysaccharides. The majority of these enzymes have not been functionally characterized, and their role in plant biomass degradation is unknown. The biotechnological challenge is to select the right set of enzymes to efficiently degrade a particular biomass. This study describes a strategy using sexual crossing and screening with the thermophilic fungus *Myceliophthora heterothallica* to identify specific enzymes associated with improved sugar beet pulp saccharification.

**Results:**

Two genetically diverse *M. heterothallica* strains CBS 203.75 and CBS 663.74 were used to generate progenies with improved growth on sugar beet pulp. One progeny, named SBP.F1.2.11, had a different genetic pattern from the parental strains and had improved saccharification activity after the growth on 3 % sugar beet pulp. The improved SBP saccharification was not explained by altered activities of the major (hemi-)cellulases. Exo-proteome analysis of progeny and parental strains after 7-day growth on sugar beet pulp showed that only 17 of the 133 secreted CAZy enzymes were more abundant in progeny SBP.F1.2.11. Particularly one enzyme belonging to the carbohydrate esterase family 5 (CE5) was more abundant in SBP.F1.2.11. This CE5-CBM1 enzyme, named as Axe1, was phylogenetically related to acetyl xylan esterases. Biochemical characterization of Axe1 confirmed de-acetylation activity with optimal activities at 75–85 °C and pH 5.5–6.0. Supplementing Axe1 to CBS 203.75 enzyme set improved release of xylose and glucose from sugar beet pulp.

**Conclusions:**

This study identified beneficial enzymes for sugar beet pulp saccharification by selecting progeny with improved growth on this particular substrate. Saccharification of sugar beet pulp was improved by supplementing enzyme mixtures with a previously uncharacterized CE5-CBM1 acetyl xylan esterase. This shows that sexual crossing and selection of *M. heterothallica* are the successful strategy to improve the composition of enzyme mixtures for efficient plant biomass degradation.

**Electronic supplementary material:**

The online version of this article (doi:10.1186/s13068-016-0460-y) contains supplementary material, which is available to authorized users.

## Background

Plant biomass has an intricate composition of different polysaccharides. For that reason, enzymatic degradation of plant biomass requires a complex mixture of enzymes. Cellulolytic enzymes are at the core of plant-degrading mixtures containing different endoglucanases, cellobiohydrolases and β-glucosidases [1]. These cellulases benefit from the presence of lytic polysaccharide monooxygenases or expansin-like proteins, which attack the crystalline parts of cellulose [2, 3]. Moreover, cellulose degradation benefits from esterases or laccases, which breaks the physical interaction between lignin and polysaccharides [4, 5]. Other examples of enzymes improving biomass saccharification are xylanases, arabinanases and pectinases [6, 7]. Identifying the essential enzymes for complete biomass degradation is often a trial and error method by adding individual enzymes to a core enzyme set [8]. Furthermore, the role of many carbohydrate-active enzymes (CAZy) is unknown, which leaves the majority of enzymes encoded on microbial genomes unexplored.

The filamentous fungus *Myceliophthora thermophila* (synonym *Sporotrichum thermophile*/*Chrysosporium lucknowense*) has shown to efficiently degrade biomass using thermostable enzymes and has more than 200 plant biomass-degrading enzymes encoded in its genome [9, 10]. These glycoside hydrolases (GH), polysaccharide lyases (PL), carbohydrate esterases (CE) and oxidases are covering most of the recognized fungal CAZy families [10]. *M. thermophila* is especially rich in enzymes with auxiliary activity (AA) with 22 genes belonging to CAZy family AA9 [10]. Even though the largest fraction of enzymes has only a putative function, many enzymes of *M. thermophila* have already been characterized in relation to polysaccharide degradation [11]. Moreover, Kolbusz and colleagues showed that 138 CAZy enzymes were detected in the exo-proteome when grown on a large range of plant biomasses [12]. Of these, 59 enzymes were important for growth on untreated plant biomass. This core set contained many cellulolytic enzymes such as nine AA9 monooxygenases, four GH1 and GH3 β-glucosidases, four GH6 and GH7 cellobiohydrolases, and two GH5 and GH45 endoglucanases [12]. This study is also a good example that growth on a particular biomass induces expression of specific enzymes such as esterase, xylanases and pectinases. However, a comparative study will be difficult for identifying the contribution of specific enzymes in releasing sugars from plant biomass.

The thermophile *Myceliophthora heterothallica* is very similar to *M. thermophila* in its morphology, physiology and phylogeny. The largest difference between the two species is the functional mating cycle of *M. heterothallica* [13]. After 2–3 weeks at 30 °C, *M. heterothallica* isolates of opposite mating type form ascomata containing dark brown ascospores at their contact zone [14]. Interestingly, progeny of *M. heterothallica* showed to have diverse physiology with respect to biomass saccharification [9]. The ability of *M. heterothallica* to sexual reproduce essentially allows selection of progeny with improved characteristics. Sexual crossing was already previously shown to be a potentially interesting strategy to improve industrial properties of filamentous fungi [15, 16].

In the current study, *M. heterothallica* isolates CBS 203.75 and CBS 663.74 were used to produce progeny with improved growth and saccharification of sugar beet pulp (SBP). Even though these isolates have the ability to produce ascomata with viable ascospores, they are genetically very different [9]. The selection of progeny based on improved growth will select for a specialist in releasing sugars from SBP. Subsequent saccharification analysis will further select for progeny with an improved enzyme set. To identify the essential enzymes for efficient degradation of SBP, parents and progeny will be compared with respect to the extracellular proteins they produced. This study shows that combining crossing and screening with genome-wide analysis are the effective strategy to identify novel enzymes that can improve saccharification of plant biomass.

## Results and discussion

### Sexual crosses between *M. heterothallica* CBS 203.75 and CBS 663.74 combined with growth screening select for strains with improved SBP saccharification

**Fig. 1:**
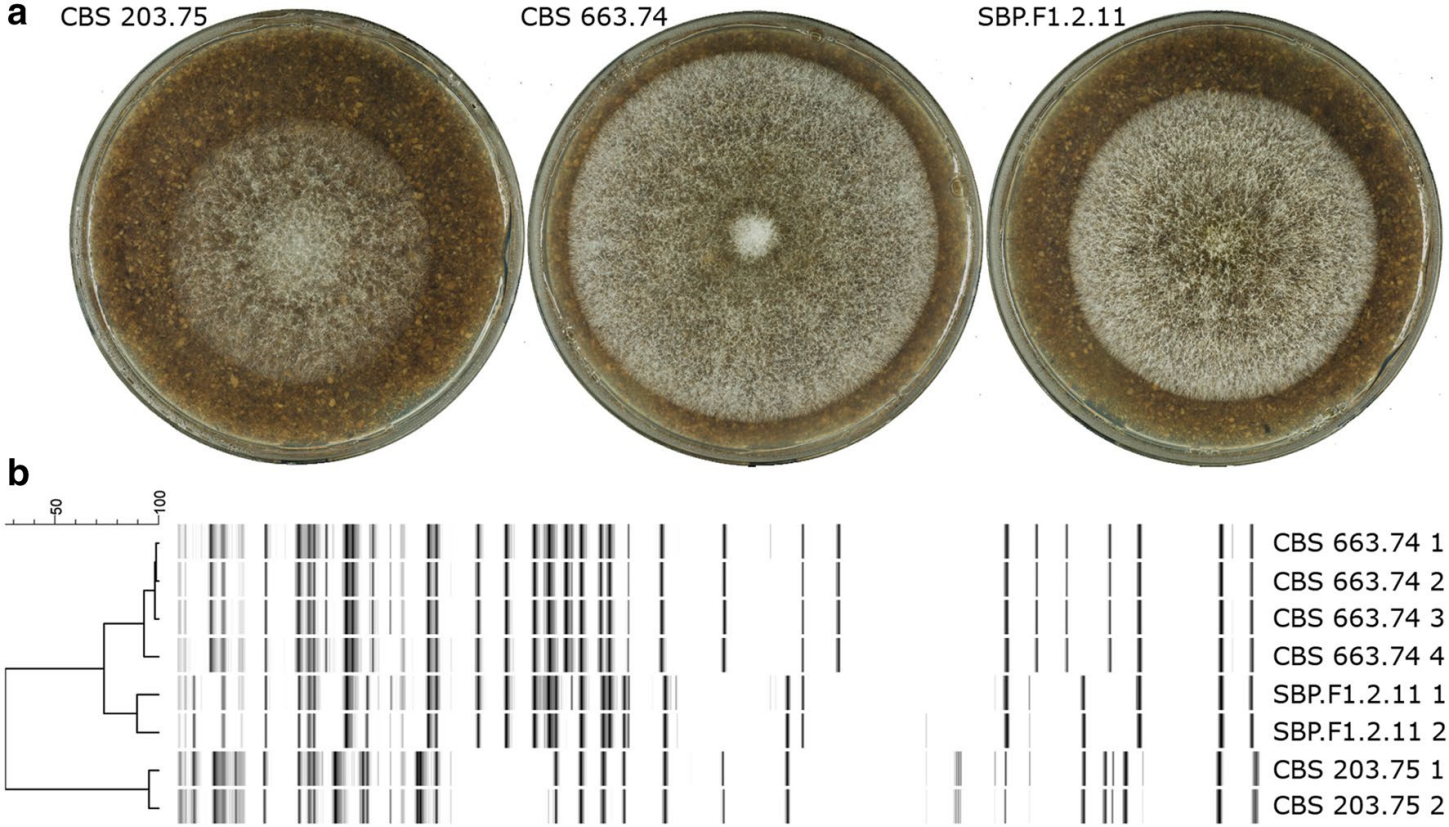
Growth on SBP (**a**) and AFLP genotyping **(b)** of parental strains CBS 203.75 and CBS 663.74, and progeny SBP.F1.2.11. The pictures are representative for colonies grown on media with 3 % SBP for 3 days at 45 °C. The similarity between AFLP banding patterns after hierarchical clustering (UPGMA) is given in percentage

The ascospores of *M. heterothallica* isolates CBS 203.75 and CBS 663.74 were plated on agar medium with 3 % SBP. After 3 days of growth at 45 °C, colonies with different sizes and densities were observed (exemplified in Fig. 1a). 70 colonies with good growth were isolated to further analyse their saccharification activity against SBP. After growing the 70 progenies and parents in 2 ml liquid SBP medium, culture supernatants were incubated for 4 h with a buffered SBP solution (pH 5.0) before measuring the total released sugar. A large fraction of progenies had improved saccharification activities compared to parental strains CBS 203.75 and CBS 663.74 (Additional file 1: Figure S1). Remarkably, 69 of the 70 progenies had the same mating type as CBS 663.74. This might be explained by interference of some germinating conidiospores of CBS 663.74, as CBS 663.74 showed better growth on SBP and higher saccharification activities compared to CBS 203.75 (15.5 ± 0.6 and 11.3 ± 0.7 µg min^−1^ ml^−1^, respectively). Still, one progeny with the mating type of CBS 203.75 had a saccharification activity of 20.5 ± 2.5 µg min^−1^ ml^−1^, which is respectively 1.8-fold and 1.3-fold higher than CBS 203.75 and CBS 663.74. Besides a higher saccharification activity, the progeny with the mating type of CBS 203.75, named SBP.F1.2.11, had also a denser colony compared to both parental strains (Fig. 1a). The genetic variation of parents and progenies was tested by amplified fragment length polymorphism (AFLP). All progenies with the mating type of CBS 663.74 had a similar AFLP banding pattern as CBS 663.74 (data not shown). However, progeny SBP.F1.2.11 had a mixed AFLP pattern of both parental strains proofing genetic content of both parents (Fig. 1b).

### Progeny SBP.F1.2.11 had improved SBP saccharification in the late phase of growth

**Fig. 2:**
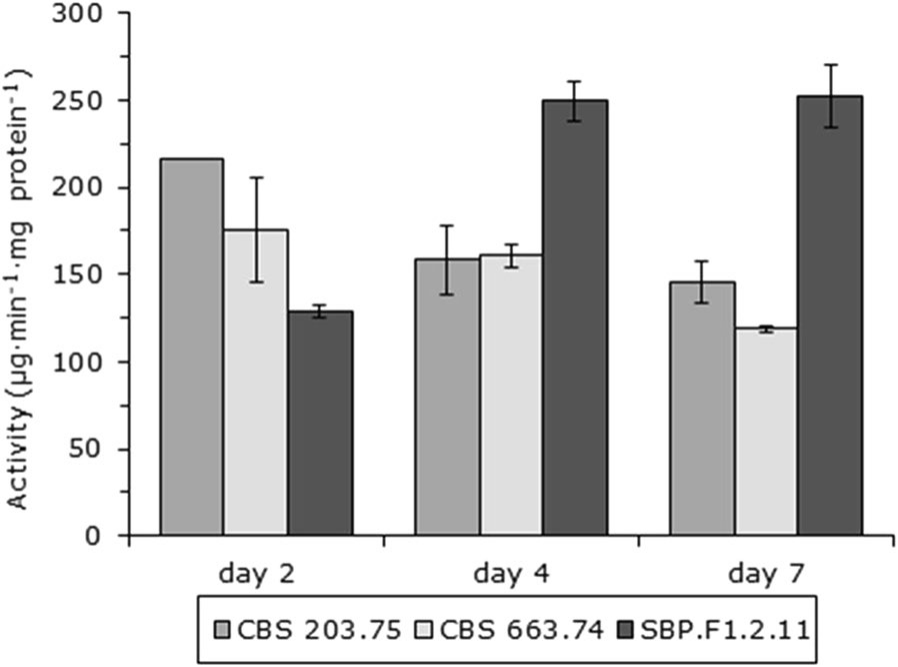
Saccharification activities against SBP of CBS 203.75, CBS 663.74 and SBP.F1.2.11 after growth on SBP for 2, 4 and 7 days. The saccharification activities were determined by the amount of reduced sugar released. The averages and standard deviations represent two independent cultivations and at least four technical replicates

Before further analysis of SBP.F1.2.11, the progeny was ten times sequentially transferred on non-selective media to ensure the genomic stability of the strain. Subsequently, progeny SBP.F1.2.11 and parents CBS 203.75 and CBS 663.74 were analysed in more detail for their saccharification activity after 2, 4 and 7 days growth on 3 % SBP (Fig. 2). Overall, saccharification activities of CBS 203.75 and CBS 663.74 decreased over the culture time, while the activities of SBP.F1.2.11 increased during cultivation. After 2 days, CBS 203.75 and CBS 663.74 had activities of 216 ± 2 and 175 ± 30 µg min^−1^ mg protein^−1^, respectively. After 7 days, these activities decreased to 145 ± 12 and 118 ± 2 µg min^−1^ mg protein^−1^, respectively. Progeny SBP.F1.2.11 had an activity of 129 ± 3 µg min^−1^ mg protein^−1^ after 2 days, and then this activity increased to 252 ± 18 µg min^−1^ mg protein^−1^ after 7 days growth. High-performance anion exchange chromatography was performed to test the composition of the released sugars. In all saccharification samples, more than 90 % of the released sugar was glucose.

### Improved SBP saccharification of progeny was not explained by an increased (hemi-)cellulase activity

**Fig. 3:**
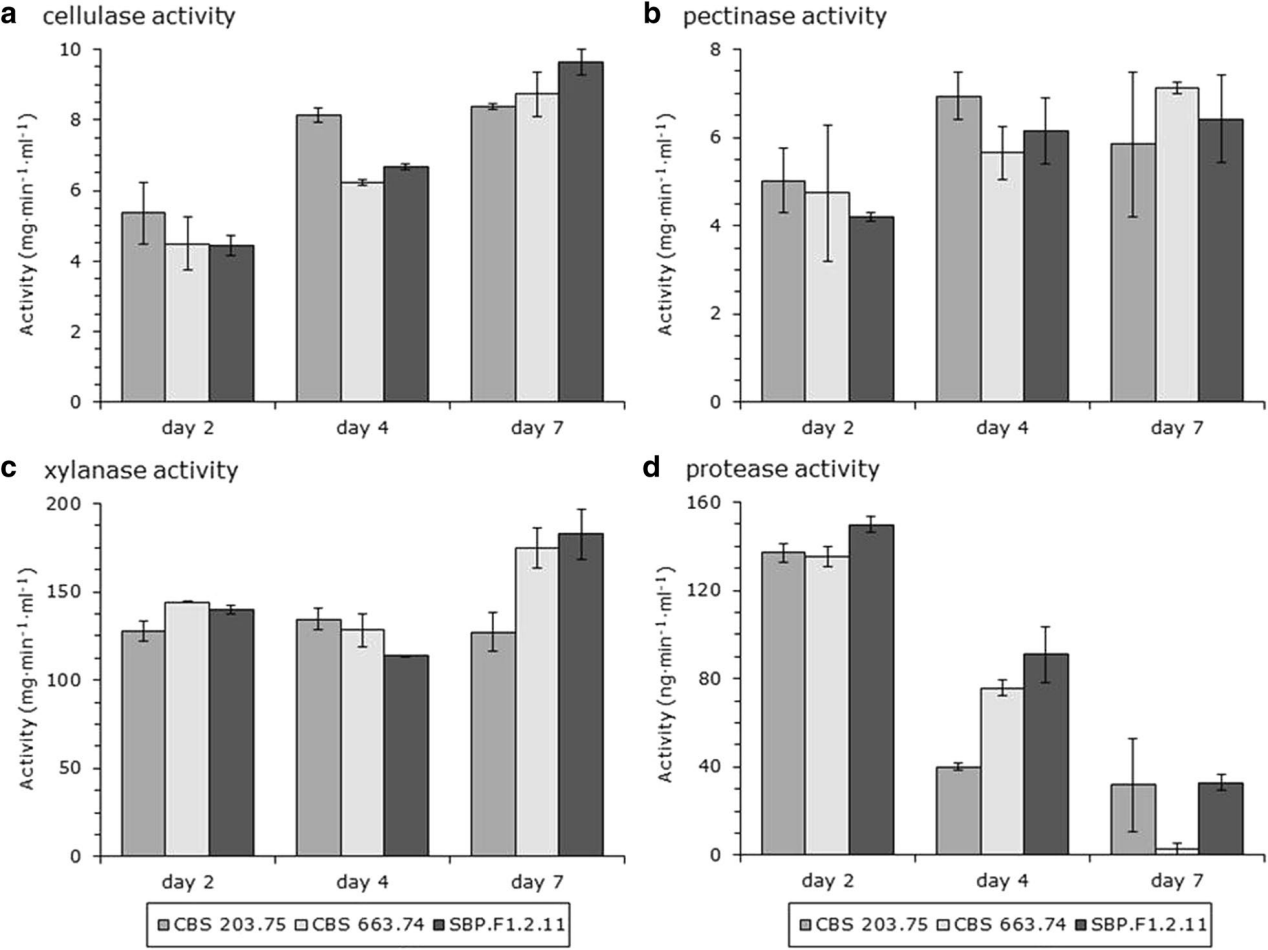
Enzyme activities of CBS 203.75, CBS 663.74 and SBP.F1.2.11 after growth on SBP for 2, 4 and 7 days. The cellulase (**a**), pectinase (**b**) and xylanase (**c**) activities were determined by the amount of reduced sugar released, while the protease activity (**d**) was determined by digestion of fluorescent-labelled casein. The averages and standard deviations represent two independent cultivations and at least four technical replicates

The large differences in SBP saccharification might be explained by altered activities of the major (hemi-)cellulases. SBP is mostly composed of cellulose and pectin structures, but also xylose-containing structures (e.g. xylan and xyloglucan) [7]. For this reason, overall activities against cellulose, pectin and xylan substrates were measured after 2, 4 and 7 days of growth on 3 % SBP (Fig. 3). Cellulase activities, as represented by activity against carboxymethyl cellulose, were not as different as the saccharification activities (Fig. 3a). The highest activities were observed after 7 days growth with small difference between parents and progeny SBP.F1.2.11. The highest activity was measured in SBP.F1.2.11 and 9.6 ± 0.4 mg min^−1^ ml^−1^, and the lowest activity in CBS 203.75 and 8.4 ± 0.1 mg min^−1^ ml^−1^. Pectinase activities, measured against apple pectin, had no significant differences between strains with the highest activities after 4 and 7 days growth (Fig. 3b). Xylanase activities, as represented by activity against beechwood xylan, were the highest for CBS 663.74 and SBP.F1.2.11 after 7 days growth (Fig. 3c). SBP.F1.2.11 had the highest activity with 183 ± 14 mg min^−1^ ml^−1^, which was 1.4-fold higher than CBS 203.75 with 127 ± 11 mg min^−1^ ml^−1^. Although these activities did not directly explain the higher saccharification activities, cellulolytic and xylanolytic activities were the highest for SBP.F1.2.11 after 7 days growth.

Another explanation for much higher saccharification activities of SBP.F1.2.11 after 7 days growth might be differences in protein turnover. As represented by activity against fluorescent-labelled casein, protease activities were highest after 2 days cultivation and decreased strongly over the cultivation time (Fig. 3d). This might be counterintuitive as nutrient limitation is likely to occur at the later phase of cultivation, which is known to stimulate protease activity [17]. Protease activity has also been linked to the extracellular pH of cultivation [18]. The extracellular pH of the cultivations increased gradually during the cultivation with pH 5.5 at day 2, pH 6.5 at day 4 and pH 7.5 at day 7. Like the measured enzyme activities, the extracellular pH did not explain the differences in saccharification activities as pH were similar between strains.

### Proteomic data identified a small cluster of proteins with higher abundance in SBP.F1.2.11

Analysing the proteome of culture filtrates will help to understand the differences in saccharification activities between SBP.F1.2.11 and parents. Before analysing their exo-proteome, the genomes of SBP.F1.2.11 and parents were sequenced, assembled and annotated. The genomes of these *M. heterothallica* strains were comparable to the genome of related species *M. thermophila* in G + C content, size and number of genes (Table 1). As the genome of *M. thermophila* was sequenced from telomere-to-telomere, the genomes of *M. heterothallica* were expected to be a bit smaller. The smaller genome and lower gene number of the assembly of CBS 663.74 and SBP.F1.2.11 could be explained by lower sequence coverage compared to the CBS 203.75 genome. Even so, the number of CAZy proteins was similar between *M. thermophila* and the three *M. heterothallica* strains. Thirteen of the 343 CAZy proteins of *M. thermophila* were not found in *M. heterothallica*. Only two of those proteins were shown to be expressed in *M. thermophila*: Mycth_90594 belonging to PL1 family and Mycth_52514 belonging to GH5 [12]. The similarity between *M. heterothallica* and *M. thermophila* was also indicated by the average sequence similarity of 96 % between CAZy proteins of both species (Additional file 2: Table S1). The most remarkable difference between *M. heterothallica* strains was the absence of two (of the three) CE5 family proteins in CBS 663.74.

**Table 1:** Genome and CAZy information of *Myceliophthora heterothallica*

	*M. thermophila* ATCC 42464 [10]	*M. heterothallica* CBS 203.75	*M. heterothallica* CBS 663.74	*M. heterothallica* SBP.F1.2.11
G + C content, %	51.4	52.5	55.9	55.6
Genome size	38.7 Mb	35.4 Mb	29 Mb	29 Mb
No. of genes	9813	10,061	8240	8322
No. of CAZy genes	343	327	327	328
GH family	186	179	180	179
PL family	8	7	7	7
CE family	27	25	23	25
AA9 family	23	22	22	22

The exo-proteome was analysed using iTRAQ technology coupled with LC–MS/MS. A total of 133 CAZy proteins were identified in culture filtrates of the three strains after 7 days growth on SBP (Additional file 2: Table S3). This number of proteins is very similar to previous exo-proteome analysis with *M. thermophila* under a wide range of conditions [12]. This study in *M. thermophila* identified 138 CAZy proteins after 48 h growth on 11 different plant biomasses [12]. Actually, 110 CAZy proteins were identified in both *M. heterothallica* and *M. thermophila*, which emphasize the similarity between both species.

**Fig. 4:**
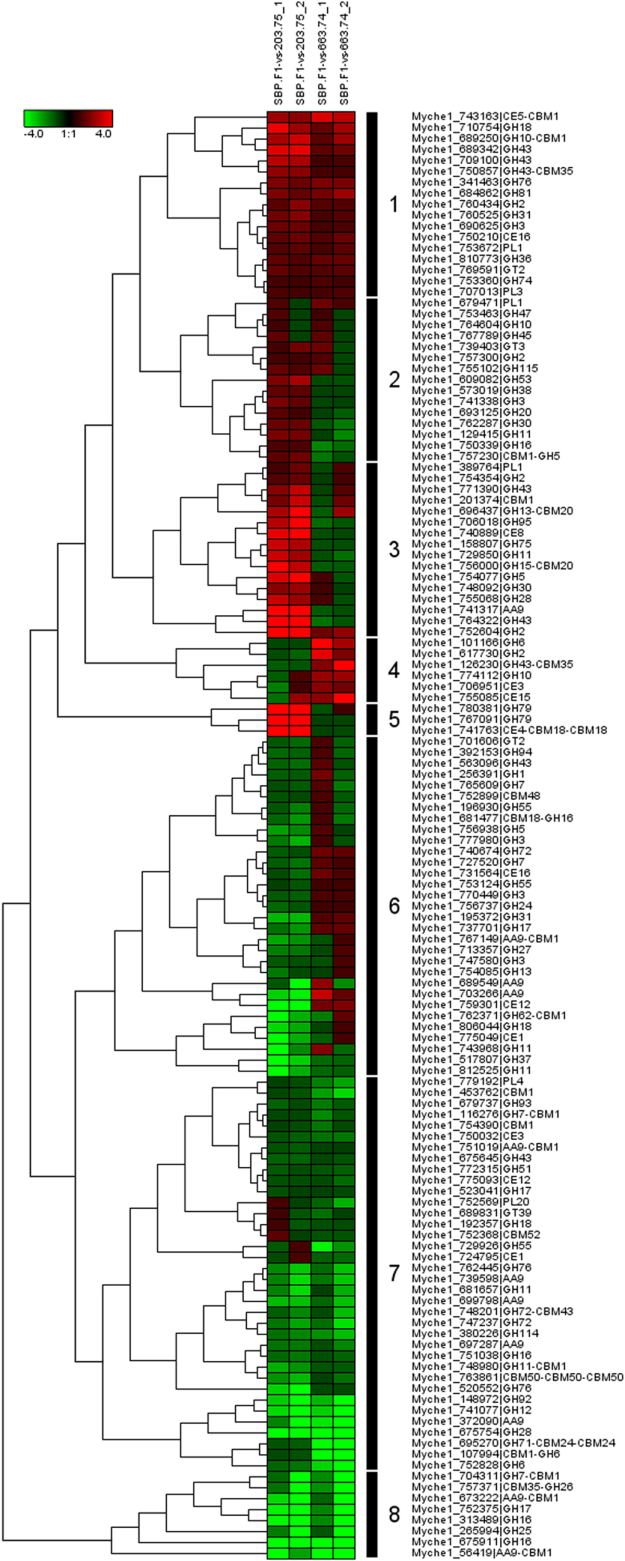
Hierarchical clustering of the 133 differentially expressed CAZy proteins between CBS 203.75, CBS 663.74 and SBP.F.1.2.11. The first two columns of the dendrogram display ratios between CAZymes of SBP.F.1.2.11 and CBS 203.75, while the last two columns of the dendrogram display ratios between CAZymes of SBP.F.1.2.11 and CBS 663.74. Each of the two columns represents proteomics data of an independent cultivation. *Green spectra* indicate proteins with decreased ratios, whereas *red spectra* indicate proteins with increased ratios between SBP.F.1.2.11 and the parental strain. The data can be found in Additional file 1: Table S3

The 133 CAZy proteins were grouped in eight clusters based on the ratio between SBP.F1.2.11 and parental strains CBS 203.75 and CBS 663.74 (Fig. 4). One-third of the proteins had a lower abundance in SBP.F1.2.11 compared to both parents (Clusters 7 and 8, 45 proteins). These clusters contained 8 of the 12 detected putative lytic polysaccharide monooxygenases belonging to the AA9 family [2]. Moreover, five putative cellobiohydrolases and endoglucanases of GH6, GH7 and GH12 family were present in clusters 7 and 8 [11]. Another large group of 31 proteins had a lower abundance in SBP.F1.2.11 compared to CBS 203.75 (Cluster 6). This cluster contained two other AA9 proteins and two GH7 proteins. Also four putative β-glucosidases of GH1 and GH3 family were present in cluster 6. Clearly, enzymes involved in cellulose degradation were less abundant in progeny SBP.F1.2.11, which is not reflected by the measured cellulase activity against carboxymethyl cellulose. Clusters 3 and 5 contained 19 proteins with a higher abundance in SBP.F1.2.11 compared to CBS 203.75. In particular, three proteins in cluster 5 were much more abundant in SBP.F1.2.11. The two beta-glucuronidases of GH79 in cluster 5 are putatively involved in arabinogalactan degradation [19]. Cluster 2 contained 15 proteins with a higher abundance in SBP.F1.2.11 compared to CBS 203.75; however, these differences are less significant compared Clusters 3 and 5. A small group of 6 proteins had a higher abundance in SBP.F1.2.11 compared to CBS 663.74 (Cluster 4). Of the 133 detected CAZy proteins, 17 proteins had a higher abundance in SBP.F1.2.11 compared to both parents (Cluster 1).

The 17 proteins with a higher abundance in SBP.F1.2.11 were separated in three sub-clusters (Table 2). Eleven proteins were only marginally more present in SBP.F1.2.11 (Cluster 1a). This sub-cluster contained a GH74 hydrolase with putative xyloglucanase activity, a CE16 esterase with putative de-acetylation activity and a GH31 hydrolase with putative α-xylosidase activity [20, 21]. All these activities are linked to degradation of xyloglucan structures [22]. The putative β-galactosidase activity of GH2 hydrolase can also play a role in xyloglucan degradation. Another sub-cluster of five proteins with a higher abundance in SBP.F1.2.11 were especially more present compared to CBS 203.75 (Cluster 1b). Three of those proteins belonged to the GH43 family. This family contains a wide range of activities against arabinan, arabinose, xylose and galactan residues (www.cazy.org). The remaining two proteins within this sub-cluster were a GH18 protein and a GH10 protein with a CBM1 motif. The *M. thermophila* homologue of this GH10-CBM1 protein has activity against non-substituted xylan chains [23]. Only one protein, belonging to the CE5 family, had a consistent higher abundance in SBP.F1.2.11 compared to both parents (Cluster 1c). CE5 proteins were reported to have esterase activities against cutin or acetylated xylan and were beneficial for saccharification of plant biomass [4, 24]. The CE5-CBM1 protein (Myche1_743163) was most similar to acetyl xylan esterases. Like the GH10 xylanase, this CE5 protein has a CBM1 motif, which might be beneficial for degradation of xylan structure in proximity of cellulose chains [25]. Even though only 4 % of SBP biomass was xylose residues (see “Methods” section), these xylan or xyloglucan chains might hinder enzymatic degradation of other polysaccharides such as cellulose and pectin [25].

**Table 2:** Hierarchical clustering in three smaller clusters a, b and c of the 17 more abundant CAZy proteins of SBP.F.1.2.11 compared to CBS 203.75 and CBS 663.74 (Cluster 1 within Fig. 4). The naming of the CAZy proteins refers to CBS 203.75. The data shows the fold change between SBP.F.1.2.11 and CBS 203.75, and SBP.F.1.2.11 and CBS 663.74 CAZymes. The data can also be found in Additional file 2: Table S3. Each of data point represents proteomics data of an independent cultivation

CAZy protein	CAZy module(s)	SBP_1-VS-203_1	SBP_2-VS-203_2	SBP_1-VS-663_1	SBP_2-VS-663_2
Cluster 1a					
Myche1_750210	CE16	1.44	1.63	1.24	1.63
Myche1_760434	GH2	1.39	2.10	1.30	1.29
Myche1_690625	GH3	1.67	1.63	1.07	1.17
Myche1_760525	GH31	1.72	2.06	1.09	1.31
Myche1_810773	GH36	1.48	1.24	1.56	1.57
Myche1_753360	GH74	1.11	1.01	1.20	1.04
Myche1_341463	GH76	1.95	1.65	1.92	1.88
Myche1_684862	GH81	1.95	1.53	1.80	2.43
Myche1_769591	GT2	1.55	1.21	1.27	1.40
Myche1_753672	PL1	1.28	1.44	1.08	1.41
Myche1_707013	PL3	1.20	1.06	1.12	1.23
Cluster 1b					
Myche1_689250	GH10-CBM1	2.60	3.17	1.39	2.42
Myche1_710754	GH18	3.66	2.42	1.56	2.53
Myche1_689342	GH43	3.46	3.57	1.43	1.72
Myche1_709100	GH43	2.83	2.66	1.03	1.15
Myche1_750857	GH43-CBM35	2.22	2.12	1.13	1.10
Cluster 1c					
Myche1_743163	CE5-CBM1	2.41	2.16	3.37	2.92

### Acetyl xylan esterase Axe1 improved SBP saccharification

**Fig. 5:**
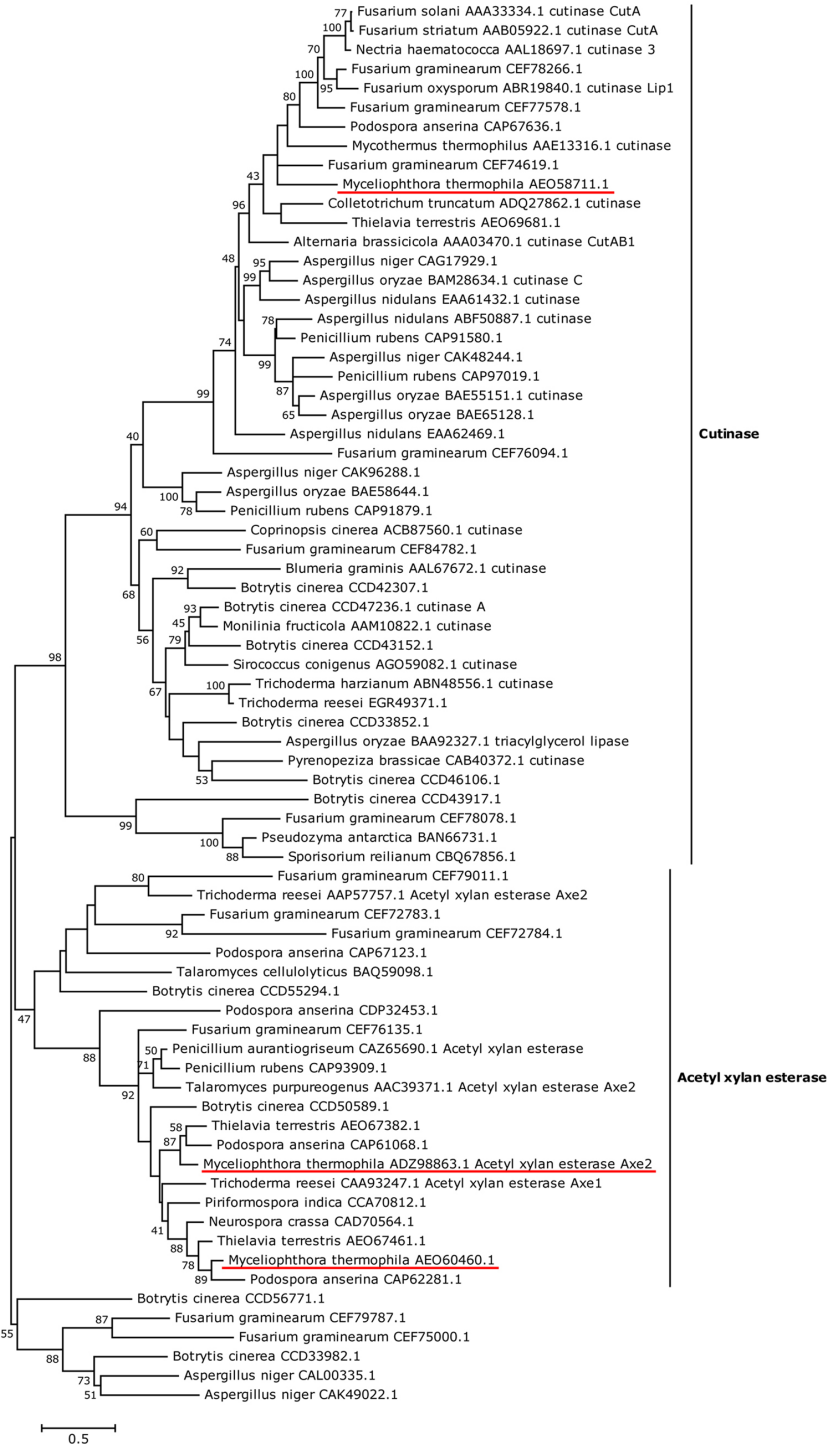
Phylogeny of fungal carbohydrate esterase family 5 proteins. Amino acids sequences were aligned, and phylogenetic tree was constructed, with MEGA software, version 5.0 [42]. Bootstrap values were calculated based on 1000 replicates of the data. The analysis using neighbour-joining method [43] involved 73 amino acid sequences of a total of 474 positions. All ambiguous positions were removed for each sequence pair. All sequences were obtained from CAZy pipeline (www.cazy.org)

To understand the significance of the proteomics results, contribution of the CE5-CBM1 enzyme in SBP saccharification needs to be analysed. First, the CE5-CBM1 protein was phylogenetically compared to 18 characterized and 54 non-characterized CE5 proteins (Fig. 5). *M. thermophila* and *M. heterothallica* have three CE5 enzymes encoded on their genomes (*M. thermophila*: AEO58711.1, ADZ98863.1 and AEO60460.1). Remarkably, *M. heterothallica* strain CBS 663.74 is missing two CE5 esterases, and has only the CE5-CBM1 enzyme encoded on its genome. AEO58711.1 from *M. thermophila* clustered together with characterized cutinases, while ADZ98863.1 and AEO60460.1 clustered with characterized acetyl xylan esterases. The ADZ98863.1 protein from *M. thermophila* ATCC 42464 is identical to the characterized acetyl xylan esterase Axe2 from *M. thermophila* C1 [26]. The *M. heterothallica* homologue of this protein (Mycth_49700) was not detected after growth on SBP. The CE5-CBM1 enzyme of our interest is AEO60460.1 in *M. thermophila* and Myche1_743163 in *M. heterothallica*. The closest characterized enzyme of AEO60460.1/Myche1_743163 is the acetyl xylan esterase CAA93247/Axe1 of *Trichoderma reesei* [27].

To test the influence of the putative CE5-CBM1 acetyl xylan esterase on the saccharification of SBP, the *M. thermophila* homologue was overproduced in a specially designed *M. thermophila* C1-expression host (LC strain) [28]. The CE5-CBM1 enzymes of *M. thermophila* and *M. heterothallica* differ on three amino acids: Thr31Ala, Ile33Val and Ala213Asp (Additional file 1: Figure S2). However, these amino acids are located in variable regions of CE5 enzymes and are far from the conserved CE5 catalytic residues Ser101, Asp188 and His200, and the region associated with substrate specificity [29, 30]. The overproduced CE5-CBM1 showed good activity against methylumbelliferyl acetate substrate with the highest activity at 75–85 °C and pH 5.5–6.0 (Additional file 1: Figure S3). Compared to the overproduced CE5-CBM1, the background proteins of the expression host had only a low activity against methylumbelliferyl acetate. Considering phylogeny and activity, the CE5-CBM1 protein of *M. thermophila* and *M. heterothallica* can be named Axe1.

**Fig. 6:**
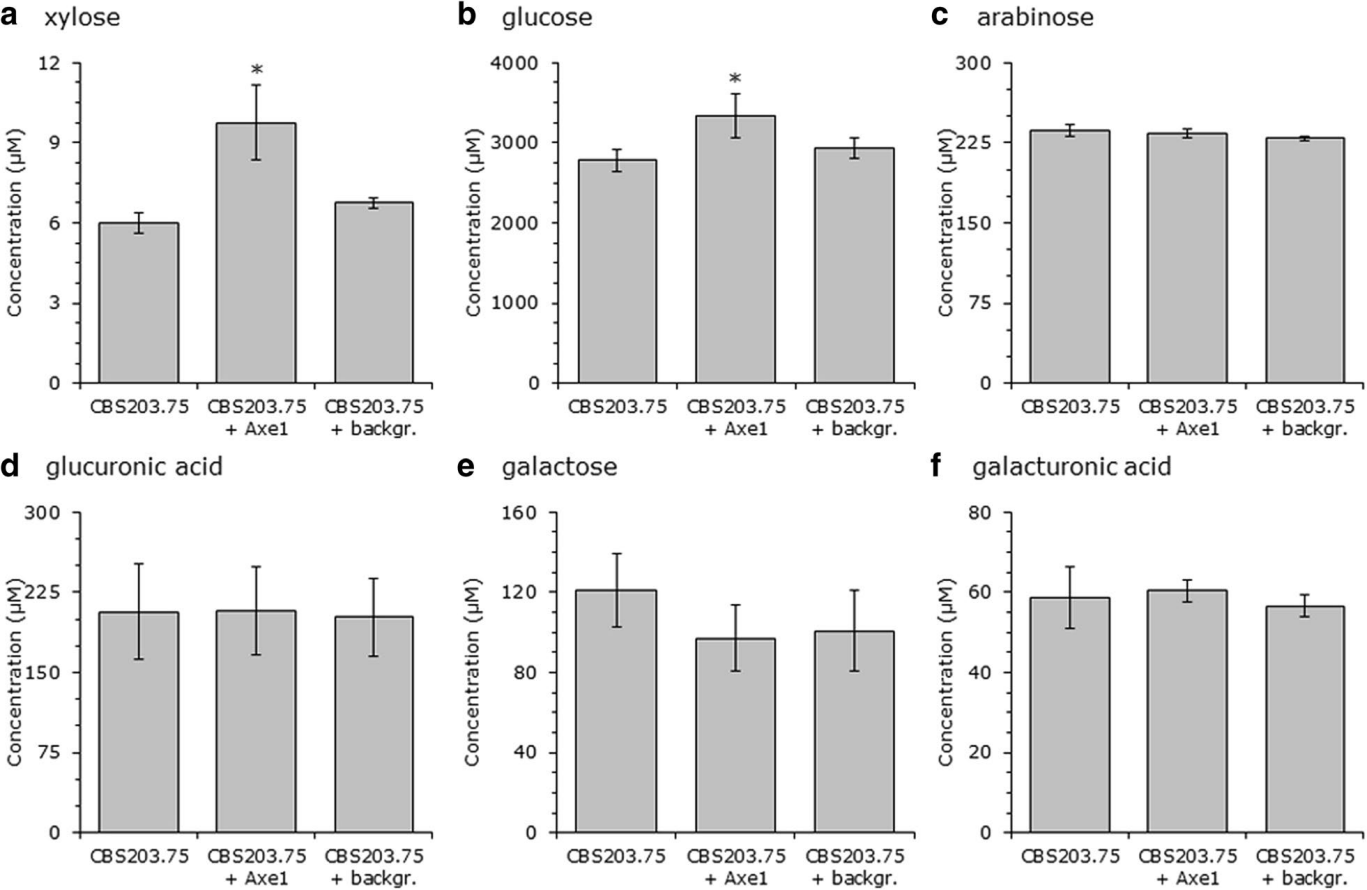
Monosaccharide release after SBP saccharification with and without CE5-CBM1 enzyme Axe1 from *M. thermophila*. Enzyme set of CBS 203.75 was obtained from a 7-day-old culture on 3 % SBP. CBS 203.75 enzyme set was supplemented with an overexpressed CE5-CBM1 enzyme Axe1 enzyme (*middle bar*) and with the background protein set from *M. thermophila* LC strain [28] (*right bar*). Monosaccharide sugars (**a** xylose, **b** glucose, **c** arabinose, **d** glucuronic acid, **e** galactose, **f** galacturonic acid) were determined by a Dionex ISC-5000 system. The averages and standard deviations represent saccharification with enzyme sets from three independent cultivations. Significance between enzyme sets with and without CE5-CBM1 enzyme Axe1 are indicated by *asterisks* (*p < 0.05)

The effect of Axe1 on SBP saccharification was tested by supplementing enzymes of a 7-day-old culture from CBS 203.75. The released monosaccharides from SBP were quantified after 24-h incubation at 50 °C (Fig. 6). The supplementation of Axe1 to CBS 203.75 enzymes significantly released more xylose with 1.6-fold increase to CBS 203.75 enzymes without Axe1 (9.8 ± 1.4 µM and 6.0 ± 0.4 µM, respectively). This positive effect on xylose release was not observed with the addition of background proteins of the expression host. The increased xylose release by supplementing Axe1 did support its role as acetyl xylan esterase. Sugars related to pectin structures, such as arabinose, galacturonic acid, galactose and glucuronic acid, did not show any significant difference between saccharification with and without Axe1. However, glucose release benefited from Axe1 supplementation with a 1.2-fold increase from 2786 ± 138 µM without Axe1 to 3340 ± 274 µM with Axe1. SBP saccharification might improve even further by adding the xylanase GH10-CBM1 or xyloglucan-related enzymes, which was identified in the exo-proteome analysis.

## Conclusions

*M. thermophila* is a well-studied thermophilic fungus and industrially applied to produce homologous and heterologous enzymes [10, 28]. Like many other industrial-relevant fungi, *M. thermophila* does not have a functional sexual cycle. Even though genetic recombination can be done parasexually, sexual crossing will greatly enhance the genetic toolbox of an industrial fungus [31, 32]. The current study used sexual crossing of the related species *M. heterothallica* to increase the knowledge on more efficient enzyme mixtures for plant biomass degradation. As both species are closely related, this knowledge obtained from *M. heterothallica* enzymes can be transferred to *M. thermophila*.

Sugar beet pulp predominantly consists of cellulose and, in lesser extent, pectin and xylan structures. Due to the complex composition of this biomass, many different kinds of enzymes might be beneficial for saccharification. This makes it immediately challenging to identify essential enzymes to further improve SBP degradation. Our strategy of combining sexual crossing and screening successfully identified a previously uncharacterized acetyl xylan esterase. The addition of Axe1 to *M. heterothallica* enzyme mixture improved the release of xylose and glucose from SBP. More knowledge on the genetics behind *Myceliophthora* mating and further optimization of the crossing strategy will make *M.heterothallica* a perfect platform for research on thermophilic fungi. In the future, the same strategy can for instance be used for a wide range of substrates to identify consistent shortcomings of *Myceliophthora* enzyme mixtures.

## Methods

### Strains and material

Strains CBS 203.75, CBS 663.74 and progeny SBP.F1.2.11 (deposited as CBS 140139) are available from the CBS-KNAW Fungal Biodiversity Centre, Utrecht, the Netherlands (www.cbs.knaw.nl).

Sugar beet pulp was mildly pretreated by exposure to 5 % hydrochloric acid (performed by GreenSugar, Germany). To remove the easily accessible sugars, the pretreated sugar beet pulp were dissolved in demineralized water, autoclaved and filtered (using filter papers, quality 4, Whatman) before using them. After this washing, the sugar composition of sugar beet pulp was approximately 49 % (w/w) cellulose, 5 % (w/w) pectin and 4 % (w/w) xylan.

### Mating

CBS 203.75 and CBS 663.74 were used as parental strains. Small agar plugs containing mycelium (1 mm diameter) from the edge of a vigorously growing 1-day-old colony of each parent were transferred to the Petri dishes with Malt Extract Agar (MEA, BD Difco) medium and were incubated in the dark at 30 °C. After two weeks, ascospores were harvested by isolating the agar with ascomata and were taken up in 5 ml 10 mM ACES (*N*-(2-acetamido)-2-aminoethanesulfonic acid) buffer, pH 6.8, supplemented with 0.02 % Tween-80 (Sigma–Aldrich, Zwijndrecht, The Netherlands). The ascospore solution was supplemented with approximately 1 cm^3^ of sterile glass beads (1:1 ratio of beads with diameters of 0.1 mm and 1.0 mm) and homogenized by vortexing for 10 min. The mycelial debris was removed by filtration through sterile glass wool. The ascospores (>1 × 10^3^ per 0.1 ml) were cultured on minimal media [33] containing 1.5 % agar and 3 % sugar beet pulp for 3 days at 45 °C.

### Screening of progeny for saccharification activity

Progeny from 70 colonies were tested for saccharification activity against sugar beet pulp. A conidiospore solution of each progeny (1 × 10^6^ spore per ml) was used to inoculate 2 ml minimal media with 3 % sugar beet pulp in a 12-well plate (Greiner, Germany). The plates were sealed with a gas permeable sealing membrane (Diversified Biotech, MA, USA). Cultures were centrifuged after 3-days cultivation at 45 °C, and supernatants were used to measure saccharification activities. 50 μl culture supernatant was added to 150 μl of 3 % sugar beet pulp in 50 mM sodium acetate (pH 5.0), and incubated for 4 h at 70 °C and 500 rpm. The total amount of released sugar was estimated using 3,5-dinitrosalicylic acid (DNS) assay [9]. All cultivations and subsequent analysis were at least done in triplicate.

### Genotyping

Mating type of progeny was identified by amplifying the mating region *mat a*-*1* and *mat A*-*3*. The *mat a*-*1* region was partially amplified using forward primer GCTTCCGCATGCAATCGTTTTT and reverse primer ACGGGTGATGTCGCTGTTAGGTAT. The *mat A*-*3* region was partially amplified using forward primer GAGAGGAAGCTGAGGGTTACG and reverse primer GTTCGCGCATTGTCAGCACT.

The overall genotype of CBS 203.75 and CBS 663.74 and their progenies was determined using Amplified Fragment Length Polymorphism (AFLP) fingerprint analysis, as described previously by Boekhout et al. [34].

### Phenotypic analysis

Strains CBS 203.75, CBS 663.74 and SBP.F1.2.11 were pre-grown on MEA medium before conidiospores were isolated in 10 mM ACES buffer. The spore solution (1 × 10^6^ spore per ml) was used to inoculate 50 ml minimal media with 3 % sugar beet pulp in a 250-ml shake flask. The cultures were grown for 7 days at 45 °C and 250 rpm, and samples were taken at days 2, 4 and 7 for physiological analysis of the culture supernatant.

The saccharification activity was measured using 50 μl culture supernatant and 150 μl of 3 % sugar beet pulp in 50 mM sodium acetate (pH 5.0). The samples were incubated in microtiter plates for 4 h at 70 °C and 500 rpm. The total amount of released sugar was estimated using 3,5-dinitrosalicylic acid (DNS) assay [9]. Furthermore, the released solubilized monosaccharides (arabinose, galactose, glucose, xylose, galacturonic acid and glucuronic acid) were quantified using high-performance anion exchange chromatography with a Dionex ISC-5000 system, which is equipped with a CarboPac PA1 (250 mm  ×  4 mm i.d.) column (Dionex, Sunnyvale, California, USA) and a pulsed amperometric detector (HPAEC-PAD).

Cellulase, pectinase and xylanase activities were determined against carboxymethyl cellulose, apple pectin and beechwood xylan (all Sigma-Aldrich, Germany), respectively. The assays contained a total volume of 200 μL using 10–50 μL of culture filtrates and 150 μL of 1 % substrate in 50 mM sodium acetate pH 5.0. The samples were incubated in microtiter plates for 30–120 min at 70 °C and 500 rpm. Subsequently, 100 μL of supernatant was mixed with 150 μL 3,5-dinitrosalicylic acid (DNS) solution. After an incubation of 25 min at 95 °C, absorbance was measured at 540 nm in a microtiter platereader (FLUOstar OPTIMA, BMG LabTech). The activities were calculated using a standard curve ranging from 0 to 2 g L^−1^ glucose. Protein concentrations were determined using Coomassie (Bradford) Protein Assay Kit (no. 23200, Pierce).

### Genome sequencing, assembly and annotation

CBS 203.75 was sequenced using Illumina technology by the Joint Genome Institute (JGI) and assembled using the standard JGI pipeline for draft fungal sequences (e.g. [10]). CBS 663.74 and SBP.F1.2.11 were also sequenced using Illumina technology, but assembly and annotation were performed in-house using ClcBio (CLC Genomics Workbench, Denmark) and Augustus v3.0.2 [35], respectively. The CAZy enzymes [36] were identified using blastp (e-value <1 × 10^−40^) against the database of *Myceliophthora thermophila* [10] (Additional file 2).

### Proteomic preparation, analysis and quantification

Quantitative proteomics were performed using iTRAQ technology coupled with 2D-nanoLC-nano-ESI-MS/MS to examine the difference of protein profiles [37, 38]. Proteins (100 μg) were digested with trypsin (Trypsin Gold, Promega, Madison, USA) at a trypsin/protein ratio of 1:20 for 4 h at 37 °C followed by another round of digestion at the same trypsin/protein ratio for 8 h at 37 °C. Digested samples were labelled with 8-plex iTRAQ reagents according to the manufacturer’s instructions (Applied Biosystems, California, USA).

The labelled samples were pooled and resolved into 12 fractions using an Ultremex SCX column containing 5-μm particles (Phenomenex, USA). The eluted fractions were then desalted using a Strata X C18 column (Phenomenex, USA) and dried under vacuum. The final average peptide concentration in each fraction was about 0.5 μg μL^−1^. Dried peptides were stored at −80 °C before MS analysis.

A splitless nanoACQuity (Waters, USA) system coupled with Triple TOF was used for analytical separation. Microfluidic traps and nanofluidic columns packed with Symmetry C18 (5 μm, 75 μm × 20 mm) were utilized for online trapping and desalting, and nanofluidic columns packed with BEH130 C18 (1.7 μm, 100 μm × 100 mm) were employed in analytical separation. Solvents purchased from Thermo Fisher Scientific (USA) were composed of water/acetonitrile/formic acid (A: 98/2/0.1 %; B: 5/95/0.1 %). A portion of a 2.25 μg (9 μL) sample was loaded, and trapping and desalting were carried out at 8 μL min^−1^ for 4 min with 99 % solvent A. At a flow rate of 300 nL min^−1^, analytical separation was established by a linear gradient from 2 to 35 % solvent B for 40 min. Following the peptide elution window, the gradient was linear increased to 80 % solvent B in 5 min and maintained for 4 min. Initial chromatographic conditions were restored in 1 min.

The peptides were subjected to nano-electrospray ionization followed by tandem mass spectrometry (MS/MS) in a Q Exactive Mass Spectrometer (ThermoFisher Scientific, San Jose, USA). The detection settings were according to [38].

The 2.3.02 version of Mascot software (Matrix Science) was used to simultaneously identify and quantify proteins. In this version, only unique peptides used for protein quantification were chosen to quantify proteins more precisely. Searches were made against the three *M. heterothallica* protein databases. Spectra from the 12 fractions were combined into one MGF (Mascot generic format) file after the raw data were loaded, and the MGF file was searched. The search parameter settings in Mascot software were according to [38].

The mass spectrometry proteomics data have been deposited to the ProteomeXchange Consortium [39] via the PRIDE partner repository with the dataset identifier PXD003130.

### Phylogeny of CE5 proteins

Fungal CE5 proteins present at the Carbohydrate-Active enZYmes Database (www.cazy.org) were downloaded and aligned with MAFFT version 7 [40]. The construction of parsimonious consensus tree was performed according to van den Brink et al. [14].

### CE5-CBM1 protein production

The CE5-CBM1 protein of *M. thermophila* C1 (identical to Mycth_2066457) was overproduced in a specially designed *M. thermophila* C1-expression host (LC strain) [28]. Both, the ‘empty’ expression host strain and the strain with the CE5-CBM1 protein were grown in fermenters of 2 litres in volume on mineral medium containing glucose, ammonium sulphate and appropriate microelements. The cells were cultivated as described in [28, 41]. The samples of the ‘empty’ expression host strain contained 13.7 % protein (w/w) and will be referred to as the background sample. The sample of the strain with the overproduced CE5-CBM1 protein contained 44.2 % protein (w/w). Approximately half of the protein in this sample were the CE5-CBM1 protein. All derived biochemical analysis of the CE5-CBM1 protein sample were compared to the background sample.

### Acetyl esterase activity

Acetyl esterase activity of the CE5-CBM1 protein was measured against 4-methylumbelliferyl acetate (M0883, Sigma–Aldrich, Zwijndrecht, The Netherlands). The assay contained 10 μL of 40 ng protein mL^−1^ sample, 10 μL of 100 μM substrates and 80 μL of 25 mM sodium acetate, pH 5.0. Change in fluorescence was measured at excitation of 360 nm and emission of 500 nm in a microtiter platereader (FLUOstar OPTIMA, BMG LabTech). Temperature optimum was determined by 5 or 10 min incubation on a heating thermomixer (Ditabis, Germany) at 30 °C till 90 °C. pH optimum was determined by a 10 min incubation at 70 °C using 0.4 M Britton–Robinson buffer in a pH range between 2 and 12.

### SBP saccharification using CE5-CBM1 protein

Strain CBS 203.75 was grown for 7 days on minimal media with 3 % SBP at 45 °C and 250 rpm. Of this culture, 1 mL of supernatant (total of approximately 262 μg) was used to incubate with 9 mL 3 % SBP in 25 mM sodium acetate, pH 5.0. Incubations were performed in 50 mL flasks at 50 °C and 250 rpm for 24 h. Part of the flasks were supplemented with 500 μL of 40 ng mL^−1^ CE5-CBM1 protein sample or background sample (total of 0.02 μg). The solubilized monosaccharides (arabinose, galactose, glucose, xylose, galacturonic acid and glucuronic acid) were quantified using a Dionex ISC-5000 system. The saccharification assays were performed with enzyme sets from three independent cultivations of CBS 203.75.

## Additional files

10.1186/s13068-016-0460-y Pdf file with three additional figures S1, S2, and S3. Figure S1 shows the saccharification activities against sugar beet pulp of 70 progenies and their *M. heterothallica* parents CBS 203.75 and CBS 663.74. The activities were measured using culture filtrates of a three-day-old culture on sugar beet pulp medium. Figure S2 shows alignment of the protein sequences of Axe1 from *M. thermophila* and *M. heterothallica*. Figure S3 shows temperature and pH profiles of the de-acetylating activity of CE5-CBM1 enzyme Axe1 of *M. thermophila* C1.
10.1186/s13068-016-0460-y Excel file with three tables S1, S2, and S3. Table S1 contains blast results of CAZy protein sequences of *M. thermophila* against the protein databases of *M. heterothallica* CBS 203.75, CBS 663.74, and SBP.F1.2.11. Table S2 contains protein sequences of CAZy proteins in *M. heterothallica* CBS 203.75, CBS 663.74, and SBP.F1.2.11. Table S3 contains the protein ratio of detected CAZy proteins between CBS 203.75 and SBP.F1.2.11, and CBS 663.74 and SBP.F1.2.11. The proteomics data have been deposited to the ProteomeXchange Consortium with the dataset identifier PXD003130.

## Abbreviations

SBP: sugar beet pulp
CAZy: carbohydrate-active enzymes
GH: glycoside hydrolases
PL: polysaccharide lyases
CE: carbohydrate esterases (CE)
AA: auxiliary activity
AFLP: amplified fragment length polymorphism
CBM: carbohydrate-binding module
