# Supplementary material for: Sexual crossing of thermophilic fungus Myceliophthora heterothallica improved enzymatic degradation of sugar beet pulp
Source: Biotechnol Biofuels. 2016 Feb 20;9:41. doi: 10.1186/s13068-016-0460-y (PMC4761134; doi:10.1186/s13068-016-0460-y)

Supplemental data

Figure S1 – Saccharification activities of 70 progenies and their parents 2

Figure S2 – Alignment of Axe1 from *M. thermophila* and *M. heterothallica* 3

Figure S3 – de-acetylating activity of Axe1 of *M. thermophila* C1 4

Figure S1.

Saccharification activities of 70 progenies of parents CBS 203.75 and CBS 663.74 against sugar beet pulp. The activities were measured using culture filtrates of a three-day old culture on sugar beet pulp medium. The dotted lines represent the saccharification activity of the parental strains. Parental strains are depicted in red and selected progeny SBP.F1.2.11 is depicted in green. The error bars represents two independent cultivations.

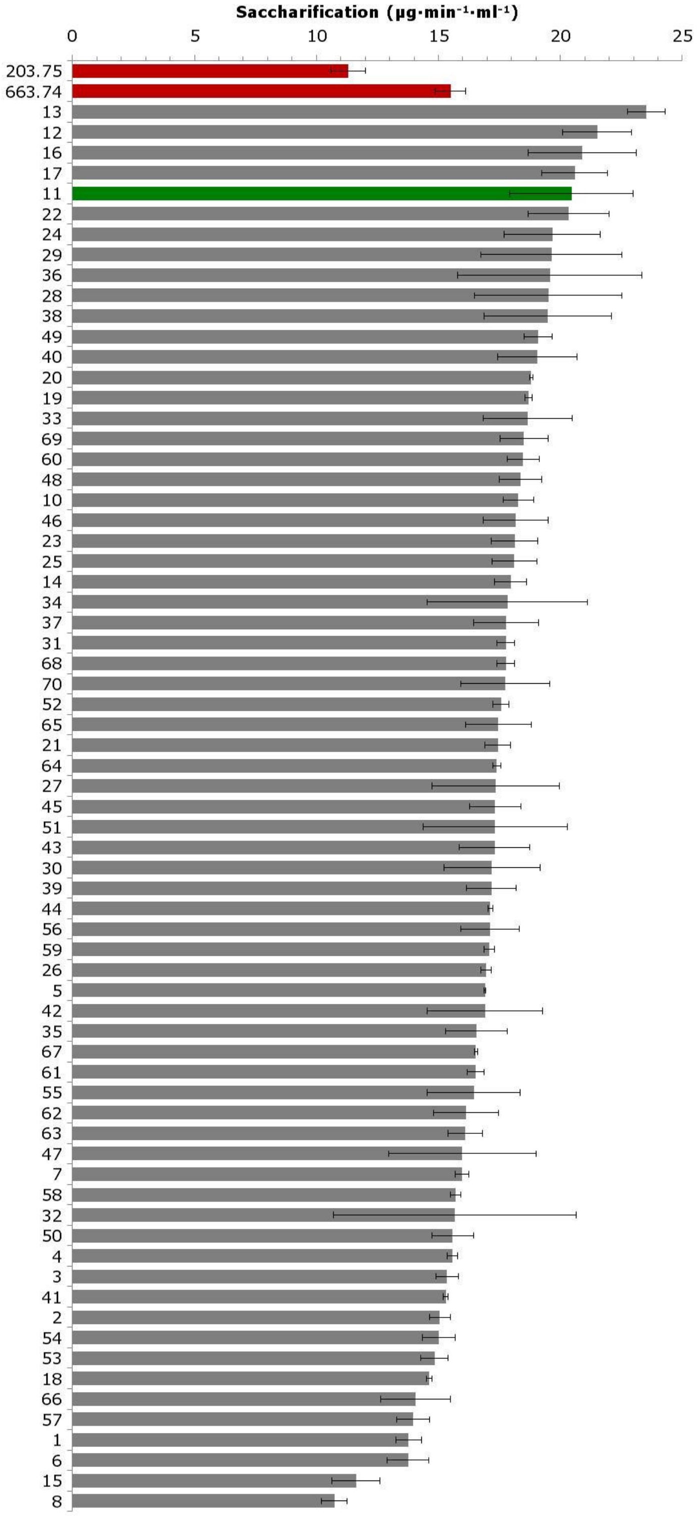

Figure S2. Protein sequence alignment of Axe1 from *M. thermophila* and *M. heterothallica*. The sequence differences between *M. thermophila* (Mycth_2066457) and *M. heterothallica* are indicated by the red arrows. The three catalytic residues are indicated by the black rounds [1], and the residues associated with substrate specificity are indicated by the black line [2].

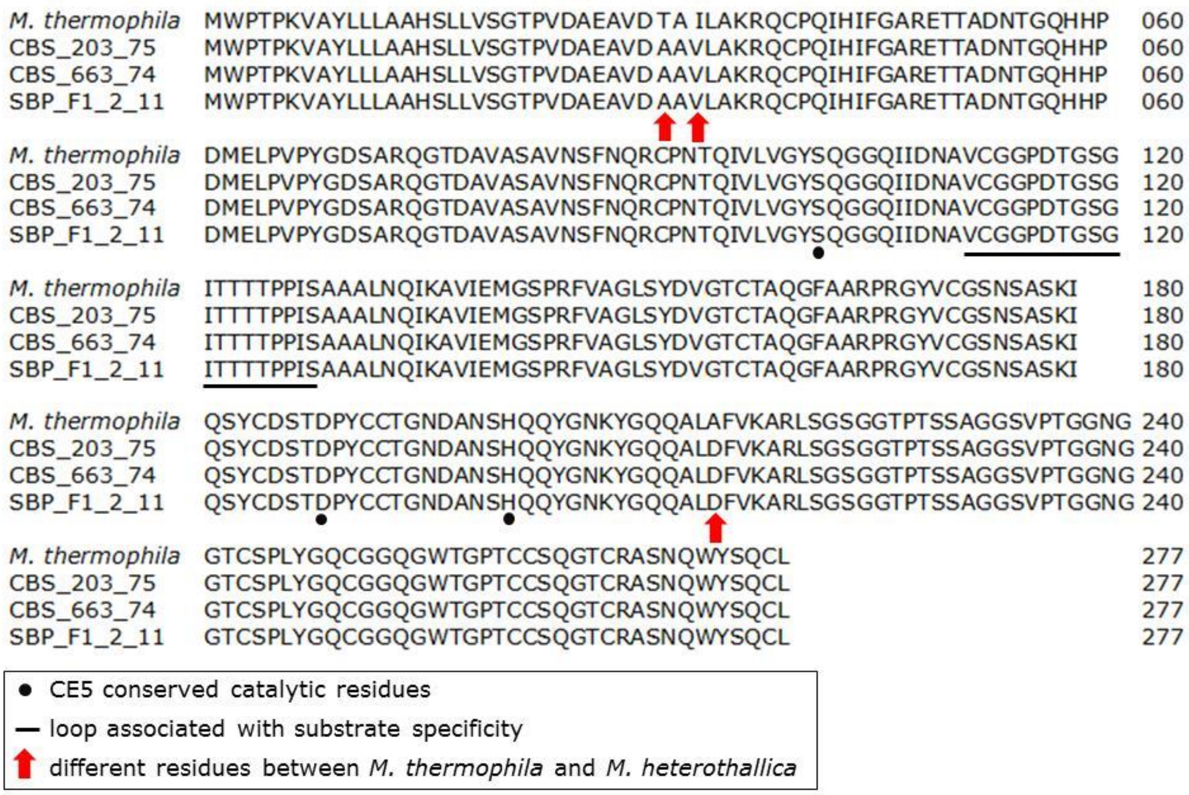

Reference

1. Hakulinen N, Tenkanen M, Rouvinen J: **Three-dimensional structure of the catalytic core of acetylxylan esterase from Trichoderma reesei: insights into the deacetylation mechanism.** *J Struct Biol* 2000, **132**:180–90.

2. Colombres M, Garate JA, Lagos CF, Araya-Secchi R, Norambuena P, Quiroz S, Larrondo L, Pérez-Acle T, Eyzaguirre J: **An eleven amino acid residue deletion expands the substrate specificity of acetyl xylan esterase II (AXE II) from Penicillium purpurogenum.** *J Comput Aided Mol Des* 2008, **22**:19–28.

Figure S3. Temperature **(A)** and pH **(B)** profile of CE5-CBM1 enzyme Axe1 of *M. thermophila* C1 (identical to Mycth_2066457) and background proteins. The activity is measured against 4-methylumbelliferyl acetate. The measurements were at least done in triplicate.

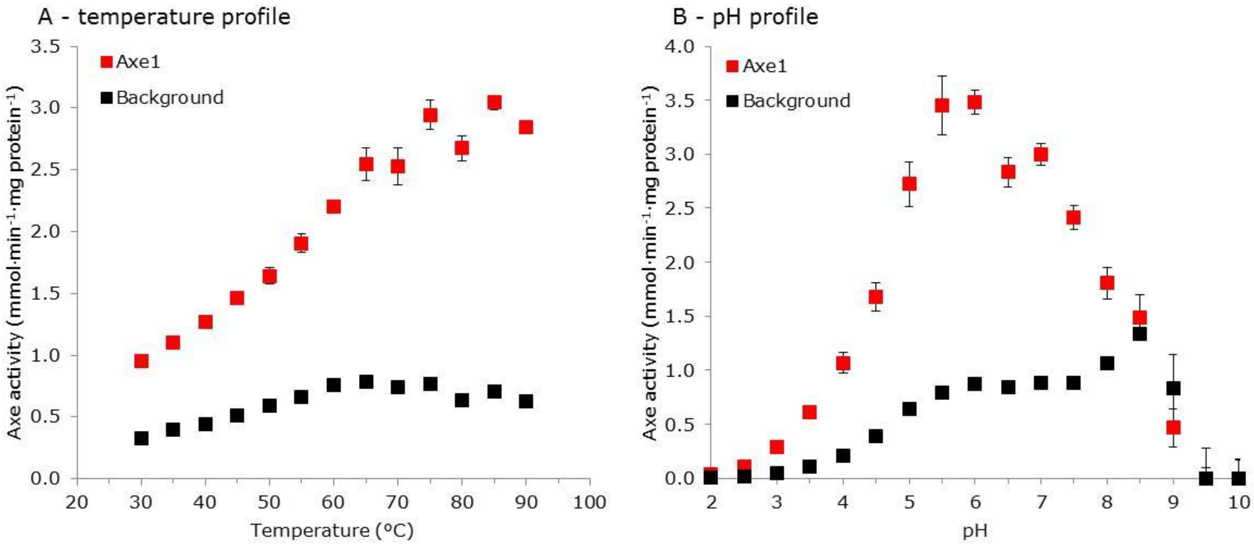

Supplement: Supplementary file 1 — 10.1186/s13068-016-0460-y Pdf file with three additional figures S1, S2, and S3. Figure S1 shows the saccharification activities against sugar beet pulp of 70 progenies and their M. heterothallica parents CBS 203.75 and CBS 663.74. The activities were measured using culture filtrates of a three-day-old culture on sugar beet pulp medium. Figure S2 shows alignment of the protein sequences of Axe1 from M. thermophila and M. heterothallica. Figure S3 shows temperature and pH profiles of the de-acetylating activity of CE5-CBM1 enzyme Axe1 of M. thermophila C1. [file 13068_2016_460_MOESM1_ESM.pdf]
